# Mass spectrometry-based metabolomic as a powerful tool to unravel the component and mechanism in TCM

**DOI:** 10.1186/s13020-025-01112-2

**Published:** 2025-05-12

**Authors:** Guang-Qin Liao, Hong-Mei Tang, Yuan-Di Yu, Li-Zhi Fu, Shuang-Jiao Li, Mai-Xun Zhu

**Affiliations:** 1 Chongqing Academy of Animal Sciences, Chongqing, 402460 China; 2 National Center of Technology Innovation for Pigs, Chongqing, 402460 China; 3 National Animal Disease-Chongqing Monitoring Station, Chongqing, 402460 China; 4 Chongqing Research Center of Veterinary Biologicals Engineering and Technology, Chongqing, 402460 China; 5 Chinese Academy of Agricultural Sciences, Beijing, 100061 China

**Keywords:** Mass spectrometry, Metabolomics, Traditional Chinese medicine, Component analysis, Mechanism

## Abstract

Mass spectrometry (MS)-based metabolomics has emerged as a transformative tool to unraveling components and their mechanisms in traditional Chinese medicine (TCM). The integration of advanced analytical platforms, such as LC–MS and GC–MS, coupled with metabolomics, has propelled the qualitative and quantitative characterization of TCM’s complex components. This review comprehensively examines the applications of MS-based metabolomics in elucidating TCM efficacy, spanning chemical composition analysis, molecular target identification, mechanism-of-action studies, and syndrome differentiation. Recent innovations in functional metabolomics, spatial metabolomics, single-cell metabolomics, and metabolic flux analysis have further expanded TCM research horizons. Artificial intelligence (AI) and bioinformatics integration offer promising avenues for overcoming analytical bottlenecks, enhancing database standardization, and driving interdisciplinary breakthroughs. However, challenges remain, including the need for improved data processing standardization, database expansion, and understanding of metabolite-gene-protein interactions. By addressing these gaps, metabolomics can bridge traditional practices and modern biomedical research, fostering global acceptance of TCM. This review highlights the synergy of advanced MS techniques, computational tools, and TCM’s holistic philosophy, presenting a forward-looking perspective on its clinical translation and internationalization.

**Supplementary Information:**

The online version contains supplementary material available at 10.1186/s13020-025-01112-2.

## Introduction

**Fig. 1:**
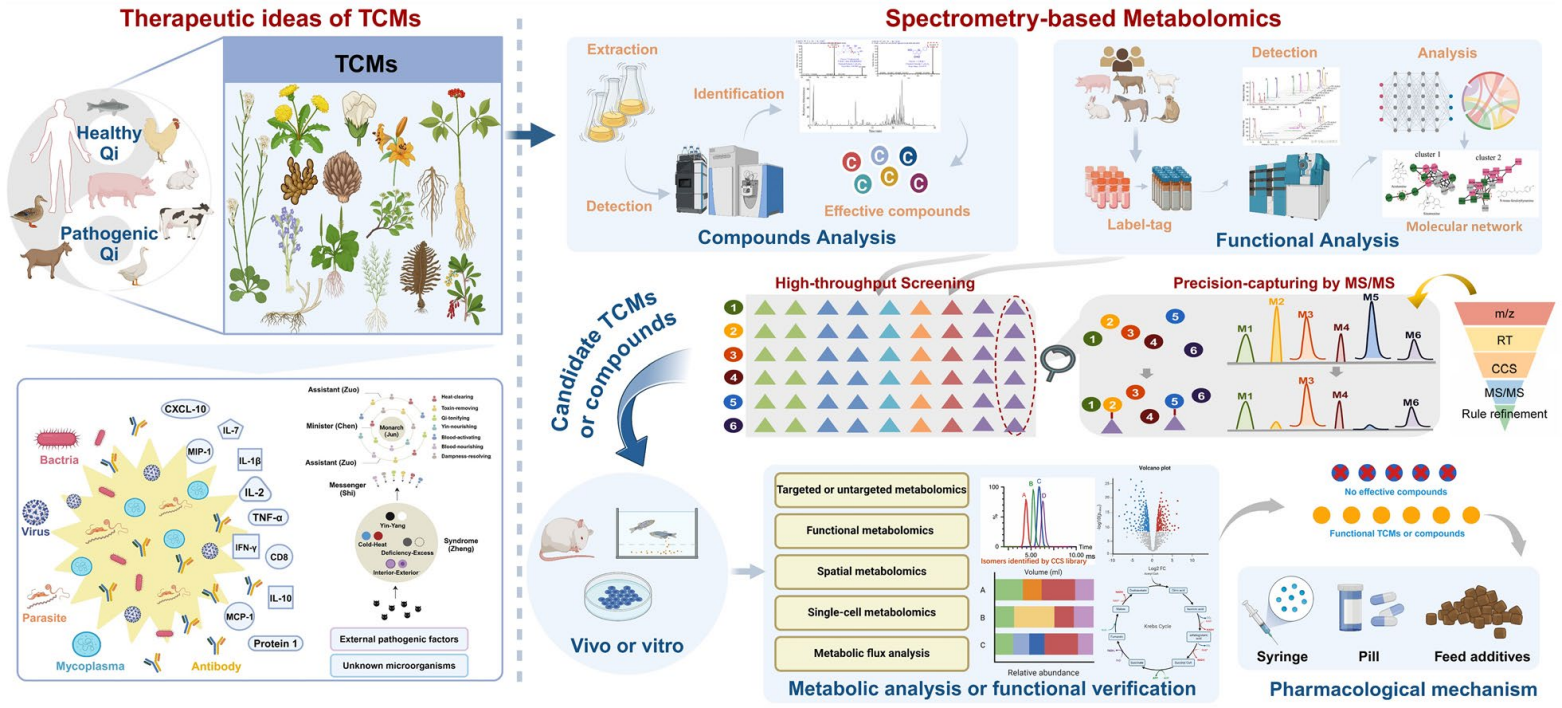
Frame of MS-based metabolomic for TCM. The exploration of TCMs and its pharmacological effects through metabolomics technology utilizing MS [1, 3, 12]

TCM has a history spanning thousands of years and holds a central role in Chinese medical practices. It demonstrates unique advantages in the prevention and treatment of various complex diseases, as evidenced by historical clinical practice [1]. Typically composed of plant mixtures with diverse chemical components, TCM exerts therapeutic effects through multi-components, multi-targets, multi-pathways, and multi-action mechanisms [2]. This approach stems from TCM philosophy, which perceives health as a harmonious balance between an individual’s internal physiological systems and the external environment. In the context of personalized treatments, TCM formulates complex herbal preparations based on the pharmacological principles of “monarch, minister, assistant, and messenger,” aiming to comprehensively regulate the body’s biochemical networks (Fig. 1) [3]. With the growing popularity of natural and holistic approaches to disease treatment, demand for TCM has been steadily increasing. The complex composition and often unspecified ingredients in TCM obscure its mechanisms of action, hindering its broader adoption, particularly in Western countries [4]. Therefore, modernizing TCM requires research into its material basis, identifying active ingredients and elucidating the underlying targets and mechanisms of action (Fig. 1). With the rise of metabolomics—a technology enabling comprehensive analysis of metabolites—TCM can now be studied with an unprecedented level of detail, offering insights into its active compounds and mechanisms.

One of the primary challenges in modernizing TCM lies in accurately identifying active components and elucidating their pharmacological properties and therapeutic effects [5]. Therefore, there is an urgent need for advanced analytical methods that can accurately characterize active ingredients and their therapeutic effects. Such methods should be capable of capturing interactions between TCM components and endogenous molecules, as well as identifying dysregulated biochemical reactions associated with diseases. Advancements in analytical techniques and systems biology have paved the way for the metabolomics, a field that leverages carefully designed experiments and data collection strategies to provide high-resolution insights into TCM’s complex chemistry [5, 6]. Metabolomics holds significant potential for streamlining drug discovery and development across multiple dimensions. Its applications range from expanding the chemical space available for activity screening to predicting molecular targets, mechanisms of action (MoA). When combined with cheminformatics, metabolomics may also suggest hypotheses about pharmacokinetic behavior (e.g., metabolic stability, bioactive metabolite generation), which can guide subsequent ADME (absorption, distribution, metabolism, and excretion)-focused studies [7–9].

MS has become a key tool in metabolomics, valued for its high sensitivity and accuracy in identifying metabolites and endogenous compounds [10, 11]. In metabolomics, high-resolution mass spectrometry (HRMS), often combined with various chromatographic techniques such as gas chromatography (GC) and liquid chromatography (LC), enhances the detection and resolution of molecule species in complex samples. HRMS is essential for resolving and characterizing the structure of compounds within complex biological mixtures [10]. Compared to basic chromatographic instruments, this integrated instrumentation provides detailed insights into TCM components, including m/z, ion fragmentation data, and collision cross sections (CCS) of ions (Fig. 1) [12, 13]. This advanced analytical capability enables a deeper understanding of TCM’s chemical complexity and paves the way for data-driven structure interpretation. The resulting data can be analyzed within the metabolic pathways through pathway enrichment analysis. It can be integrated with orthogonal data from proteomics, transcriptomics, and genomics, enabling a holistic view of TCM’s molecular interactions. Traditional molecular biology assays further contribute by elucidating molecular mechanisms and linking to TCM components to disease-associated biochemical pathways [6]. In TCM research, analyzing extensive chemical components must be aligned with assessments of pharmacological effectiveness.

Based on the aforementioned background, this review provides a comprehensive overview of MS-based metabolomics techniques and their applications in the pharmacodynamics of TCM. The review primary focuses on how chromatography and MS-based metabolomics are utilized to investigate the chemical composition, underlying biological mechanisms and syndrome of TCM. Furthermore, this review examines how metabolomics approaches contribute to pharmacological validation, and efficacy evaluation of TCM, offering a path forward in setting standardized benchmarks for its use.

## Analytical methods

**Fig. 2:**
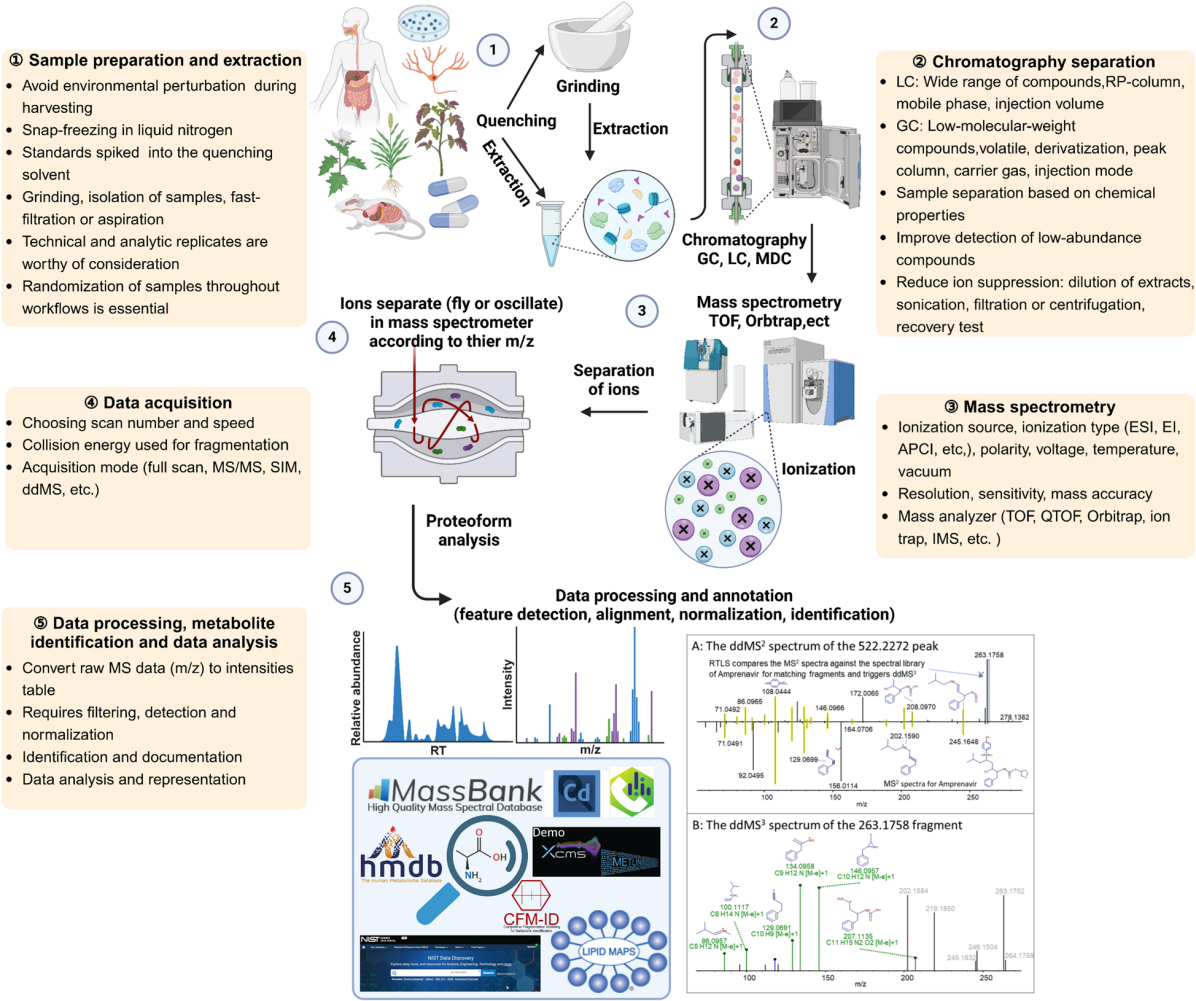
General overview and schematic content of MS-based metabolomics workflow [14–16]

In recent years, metabolomics has emerged as a leading approach to describe phenotypes and serves as a bridge between genotypes and phenotypes. Therefore, achieving sufficient coverage of metabolites in terms of their chemical structures and dynamic abundance range is crucial for understanding key biological information and bioactive metabolites. However, current technologies for metabolite qualitation and quantification remain limited in scope and comprehensiveness. To address these limitations, researchers have developed various analytical methods to expand metabolite coverage. Figure 2 illustrates the typical workflow in metabolomic fingerprinting, including sample preparation, separation techniques, and MS analysis [14–16]. Sample preparation techniques enable comprehensive metabolite extraction, while separation techniques reduce MS data complexity, and MS analysis provides detailed information across the full spectrum of metabolites. Together, these analytical techniques improve detection sensitivity and enhance the depth of metabolomic data.

### Sample preparation

Sample preparation is a crucial step prior to MS analysis to enrich target metabolites and eliminate impurities. Before sample extraction, in order to allow the extracting agent to fully contact with the sample and extract more metabolites as much as possible, it is necessary to homogenize the sample to reduce its size and also achieve quenching of metabolic reactions in the sample [16]. Common extraction methods include solid–liquid extraction (SLE) and liquid–liquid extraction (LLE) [17]. Water, methanol, and acetonitrile are typical extractants, with formic acid as an additive to cover a wide polarity range, suitable for polar and weakly polar metabolites [18]. For non-polar metabolites such as lipids and volatile compounds, non-polar solvents like dichloromethane and chloroform are used [15]. Additionally, cold solvents are often employed to reduce enzymatic activity, followed by centrifugation to remove solid residues and protein precipitates [18]. Alternative techniques like laser microdissection, microdialysis sampling, and enzyme-assisted extraction can be employed for specific analytical requirements [18, 19]. Researchers can individually choose suitable methods based on the characteristics of their target analytes according to their experimental requirements. For GC analysis, derivatization is necessary to reduce metabolite polarity, allowing the analysis of amino acids, fatty acids and other compounds [15]. These additional steps add complexity to sample handling, making GC-based metabolomics less common in general research.

### Separation techniques types coupled with MS

Following sample preparation, MS is a primary analytical platform for metabolite detection due to its sensitivity and specificity [17]. Consequently, MS-based metabolomics has become an essential force in metabolomics. However, metabolites analysis in complex biological samples often faces challenges, such as co-elution and low responsiveness [16]. Separation prior to MS detection is essential to improve peak capacity and resolution, enabling more comprehensive metabolite profiling and higher result quality [16, 20]. Therefore, coupling separation techniques with MS reduces data complexity, improves detection sensitivity and provides useful information in physical and chemical properties of the analytes. The predominant separation techniques coupled with MS include GC, LC, multidimensional chromatography (MDC), each suitable for different types of metabolite analyses [7, 10, 17, 21]. This review provides an overview of these mainstream separation techniques, focusing on their applications and limitations in metabolomics.

#### GC separation technique

GC is suited for separation thermally stable volatile compounds, including or pre-derivatized volatile derivatives [15, 22]. Common stationary phase includes non-polar dimethyl polysiloxane and polar polyethylene glycol, while high-purity helium or nitrogen serves as the mobile phase [23]. In metabolomics, 5–95% dimethyl polysiloxane and high purity helium gas are commonly used for GC separation [15, 24]. When GC coupled with MS, GC–MS offers high resolution, sensitivity and good separation reproducibility, making it suitable for detecting low concentration compounds. This technique has been widely applied in metabolomics [25]. In TCM efficacy studies, GC–MS is mainly used for analyzing volatile oils, determining TCM component content, characterizing endogenous metabolites in biological systems, and profiling internal TCM constituents [22, 24]. However, samples containing moisture require drying or solvent exchange before GC–MS analysis, which may lead to the loss of water-soluble volatile compounds [15]. Although GC was one of the first separation technique used in metabolomics, it requires derivatization and other preparatory steps to increase compound detectability, limiting its suitability for high-throughput applications. This limitation has led to a preference for LC in metabolomics.

#### LC separation technique

**Fig. 3:**
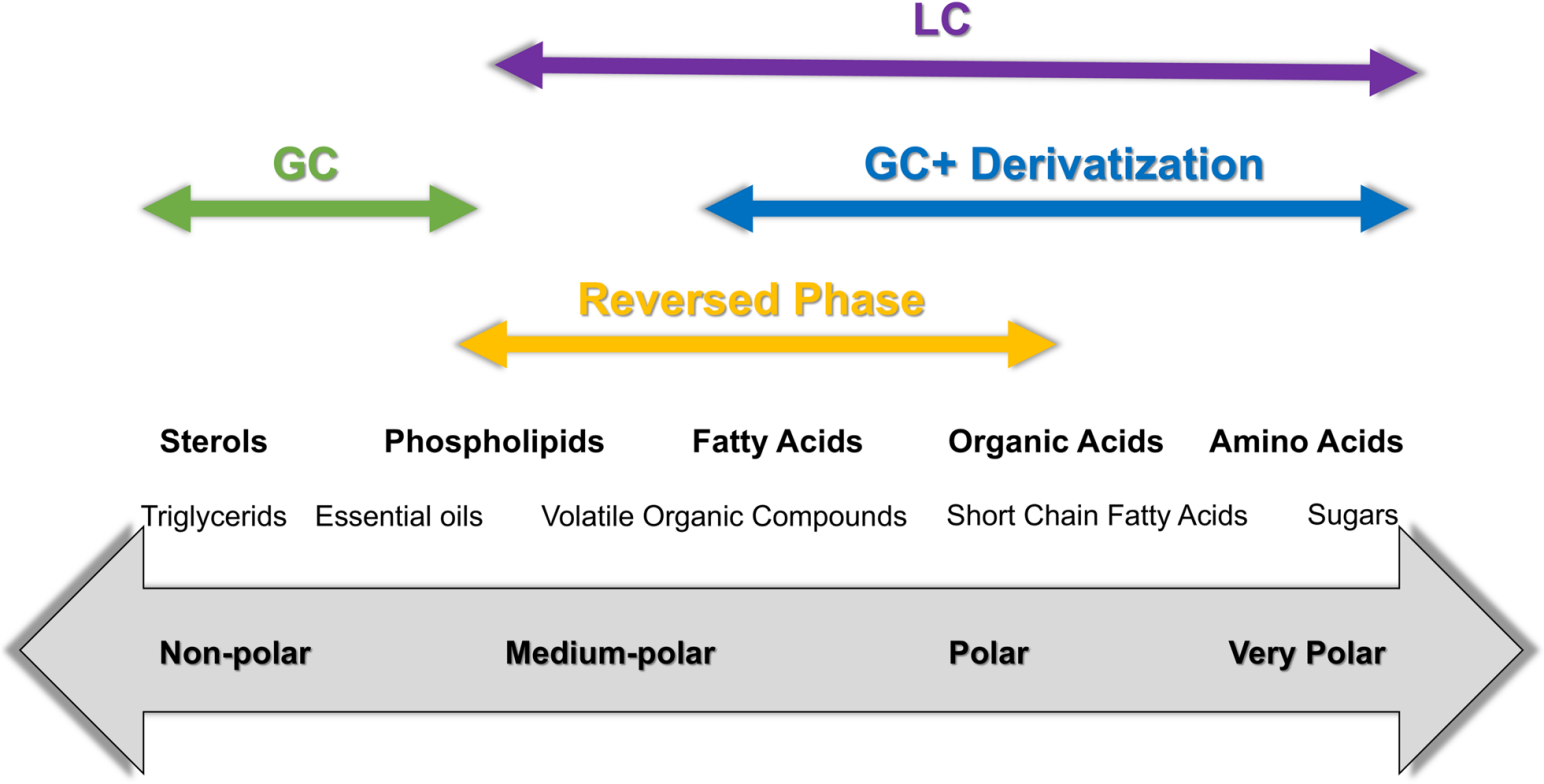
Recommended chromatography-based analysis methods of various molecular features [33]

LC is the most commonly used separation technique in MS-based metabolomics, suitable for separating polar, non-volatile, and/or thermally unstable compounds [15]. LC separates compounds based on the interaction between the stationary and mobile phase in the chromatographic column [26]. These interactions mechanisms include polarity, ion-pairing, and size exclusion [26]. Unlike GC, LC is suitable for aqueous samples and minimal sample preparation, with shorter separation times [27]. Reverse-phase liquid chromatography (RPLC) dominates metabolomics due to its robustness and compatibility with mass spectrometry. Its hydrophobic stationary phase (e.g., C18) retains non-polar to moderately polar compounds, while the aqueous-organic mobile phase (e.g., acetonitrile/water) allows gradient elution [28]. Key advantages include high reproducibility across laboratories, broad coverage of metabolites (e.g., flavonoids, lipids, semi-polar alkaloids), compatibility with ESI–MS due to low salt requirements [15]. For example, Hyun-A Oh et al. used RP-C18 to analyze plasma metabolites after herbal supplement intake, demonstrating the column's applicability in TCM studies [29]. Kozlowska et al. utilized this stationary phase for the separation and detection of organic acids and nitrogen-containing bases in urine, demonstrating its effectiveness in polar metabolite analysis [30]. However, poor retention of highly polar compounds (e.g., organic acids, sugars), necessitating complementary approaches like Hydrophilic interaction chromatography (HILIC) [15]. Traditional normal-phase (NP) chromatography uses polar stationary phases (e.g., silica) with non-polar solvents (e.g., hexane). While effective for lipid classes (e.g., triglycerides), its incompatibility with aqueous samples and MS limits metabolomics applications [18]. HILIC, a modern variant of NP, employs polar phases (e.g., amide, zwitterionic) with water-miscible organic solvents (e.g., acetonitrile + 5–40% aqueous buffer). Its advantages include strong retention of polar metabolites (e.g., amino acids, nucleotides, phosphatidylserine), and direct coupling with ESI–MS due to high organic solvent content [31]. However, sensitivity to buffer conditions (e.g., pH, ion strength), reducing inter-laboratory reproducibility compared to RPLC [15]. To achieve maximum peak capacity, combining RPLC and HILIC is recommended to maximize peak capacity, particularly in untargeted metabolomics studies [32]. Figure 3 shows the selection criteria for chromatographic methods based on compound type and polarity [33].

#### Comprehensive two-dimensional GC (GC × GC)

With the continuous development of separation technology, GC × GC is widely used for separating and characterizing complex mixtures, thus giving rise to multidimensional chromatography (MDC) [34]. GC × GC enhances chromatographic separation by connecting two capillary columns in series through a modulator, which captures, concentrates, and releases compounds from one-dimension (^1^D) column to two-dimension (^2^D) column, offering 3–10 times higher peak capacity and generating spectra with ^1^D and ^2^D retention times (^1^tR and ^2^tR) to improve MDC analysis effectiveness [35–37]. To differentiate co-effluent compounds from ^1^D, the two columns in GC × GC should have different polarities, typically using a non-polar column with gradient ramping in ^1^D and a polar column under isothermal conditions in ^2^D. To prevent modulation cycle wrap-around due to the shorter separation time in ^2^D, a 30 m column is commonly used for ^1^D, while a column shorter than 5 m is employed for ^2^D [36]. GC × GC is widely used for analyzing in vitro cultures (e.g., bacteria, cells), biological samples (e.g., blood, urine), and organisms (e.g., primates, pigs, mice, natural products) [21, 36]. Nonetheless, GC × GC has certain limitations, including the loss of ^1^D resolution when capturing target fractions and the need to re-analyze multiple fractions on both dimensions, increasing workload and complexity [37]. Therefore, GC × GC requires advanced data visualization techniques, and numerous studies focus on visualization methods to facilitate interpretation [34, 38].

#### Comprehensive two-dimensional LC (LC × LC) separation technique

Similar to GC, LC also allows multidimensional separation. In 1987, Giddings et al. proposed the concept of ^2^D LC [39]. It has been demonstrated effective for separating complex mixtures over the past few decades [38, 40]. ^2^D LC combines two LC columns and can operate in offline or online modes. Offline mode has lower automation, requiring manual reinjection of the ^1^D column effluent into the ^2^D column for further separation. Online mode automatically transfers all components from the 1D column to ^2^D column, making it suitable for high-throughput analysis [13, 41]. Furthermore, online ^2^D LC can be further classified into heart-cutting ^2^D LC (LC-LC) and comprehensive ^2^D LC (LC × LC) methods. In LC-LC method, only selected components containing the target analyte are transferred to the ^2^D column, making it suitable for targeted metabolomics. In this method, all components are separated in the ^2^D column, allowing comprehensive information collection in non-targeted analysis of complex mixtures [13, 41]. Rick S. van den Hurk et al. reviewed the latest advancements in ^2^D LC, and found that LC-LC is mainly used in pharmaceuticals and biopharma industries, whereas LC × LC is commonly applied to food and TCM separation [42].

Offline ^2^D LC avoid the problem of solvent immiscibility. The chromatographic columns used in this separation system have several combinations such as NP × RP, HILIC × RP, RP × RP, when coupled with MS detection, provide high peak capacity and excellent compatibility [13]. Combining NP with RP enhances polar compound separation, as shown by Zhang et al., who detected 64 anticancer bufadienolides in toad skin using NP × RP LC–MS [43]. HILIC × RP complements the separation of glycosides, steroids, and phenolic acids, with Yao et al. characterizing ginsenosides in *Panax notoginseng* leaves [44]. RP × RP exploits pH orthogonality, as demonstrated by Pan et al., who identified indole alkaloids from five plant sources using acidic ^1^D RP and alkaline ^2^D RP [45].

In online ^2^D LC, automation between columns is achieved through modulators, typically equipped with switching valves containing 6, 8, 10, or 12 ports. The 6-port valve is used in LC-LC to transfer target components, while other valves support both LC-LC and LC × LC, detailed transfer matrices can be found in reviews by Ji et al. and Rick S. van den Hurk et al. [41, 42]. LC-LC excels in targeted screening and purification of TCM compounds, as shown by Fang et al., who isolated antioxidant inhibitors from Saxifraga atrata using an online LC-LC system [46]. LC × LC is ideal for TCM chemical fingerprinting, with HILIC × RP commonly used for natural product analysis [47]. Solvent polarity differences in ^2^D can reduce separation efficiency, but similar column pairings like HILIC × HILIC or RP × RP effectively address this [48, 49].

### MS techniques

Metabolomics relies on multiple analytical techniques to analyze complex samples and diverse small molecules. Therefore, using multiple analysis platforms is essential to enhance compound coverage and obtain comprehensive information. Over the past few decades, MS has been the primary analytical platforms in metabolomics [7, 50]. Innovations in MS technology are driving the development of metabolomics, with a primary focus on improving mass resolution and sensitivity. Therefore, this section reviews key technical of MS, including ionization techniques, types of mass analysis, data acquisition modes, and data analysis software.

#### Ionization techniques3

Before MS analysis, metabolites must be ionized into charged ions for detection and analysis, with ionization techniques varying based on the separation technique, primarily categorized as GC–MS and LC–MS (Table S1) [10].

Electron ionization (EI) is widely used in GC–MS [10]. The EI source, consisting of a tungsten filament, ionization chamber, and lenses, generates high-energy electron beams (70 eV) that ionize gas-phase molecules into odd-electron cation radicals (M + ·), which fragment into stable ions. These ions are separated by their m/z values in the mass analyzer. EI’s consistent fragmentation patterns provide high reproducibility, enabling easy database searches in resources like NIST, Fiehn Lib, and Wiley [51, 52]. For example, Mohammad et al. discussed the application of GC-EI-MS in urine metabolomics research [53]. Annalaura Mastrangelo et al. also reviewed the application of GC-EI-MS in metabolomic analysis [54].

Electrospray ionization (ESI) is preferred in LC–MS for detecting various metabolites [15]. In ESI, a liquid sample passes through a high-voltage capillary, forming a Taylor cone that emits charged droplets. During evaporation, polar molecules ionize into gas-phase ions, which are introduced into a mass spectrometer for molecular mass and structure analysis. Ionization efficiency can be improved by adding polar solvents (e.g., water–methanol, acetonitrile) and electrolytes (e.g., formic acid, ammonium salts) [55]. In MS-based metabolomics, co-elution reduces ionization efficiency due to surface charge competition, known as the matrix effect (ME). ME can be mitigated by sample dilution, target compound extraction, and optimizing separation techniques [26, 55]. Adding salts like ammonium acetate or ammonium formate adjusts solvent pH and enhances ionization, while non-volatile salts, such as phosphates, can suppress ionization by forming interfering adducts [55, 56]. Innovations in ESI have created nano-electrospray ionization (nano-ESI) [57], induced nano-electrospray ionization (InESI) [58], electrosonic spray ionization (ESSI) [55], extractive electrospray ionization (EESI) [55], which facilitate analysis of metabolites in complex matrix. For spatial metabolomics, desorption electrospray ionization (DESI) [59] and matrix-assisted laser desorption/ionization (MALDI) [7] are widely used. Additionally, direct analysis in real-time mass spectrometry (DART-MS) utilizes atmospheric gases to directly extract ions from sample surfaces, making rapid screening and distribution analysis of target compounds in biological samples like TCM and animal tissues, this makes it highly suitable for on-site detection [10].

#### Types of mass analysis

Mass analyzer separates and detect ions based on their m/z using electric or magnetic fields. Over the past 100 years, various types of mass analyzers have been developed. Common low-resolution mass analyzers include quadrupole, triple quadrupole (QQQ), ion trap (IT), and Q-Trap. HRMS include Orbitrap, time-of-flight (TOF), Fourier transform ion cyclotron resonance (FT-ICR), ion mobility spectrometry (IMS) [22, 26, 33].

Low-resolution MS uses two analyzers connected by a collision pool for ion fragmentation. The first analyzer filters precursor ions, which are fragmented in the collision pool and analyzed by the second analyzer [26]. It excels in targeted quantification with high sensitivity and selectivity but is limited in identifying unknown compound structures [20]. For example, Ding et al. used LC-QQQ-MS/MS for quantitative analysis of 68 components in Angong Niuhuang Pil [60], while Hu et al. monitored over 250 metabolites related to cardiovascular and viral diseases using a targeted metabolomics platform [7].

HRMS offers higher quality resolution and sensitivity, making it suitable for precise analysis of molecular weights and secondary structures [22]. In HRMS, TOF, Orbitrap and IMS are widely used in metabolomics, while GC–MS, LC–MS, and IM–MS are commonly used MS-based metabolomics (Table S1). Additionally, quadrupole can combine with TOF or Orbitrap to form Q-TOF and Q-Orbitrap configurations [18, 26]. The performance of various HRMS models, as shown in Table S1, highlights that while Orbitrap offers superior resolution, its longer duty cycles limit its suitability for high-speed analyses, whereas Q-TOF, with the ability to capture up to 100 MS/MS spectra per second, excels in precise quantification of closely spaced peaks [18, 20]. Xing et al. used LC-QTOF-MS for non-targeted metabolomic analysis of TCM extracts, followed by bioactivity validation of the interested compounds [61]. Li et al. analyzed the components of Sanhuang tablets using Q-Orbitrap-MS [62].

IMS is a novel analytical method that separates ions based on their size and shape in the gas phase. Under an electric field, ions move through an inert gas, and differences in mobility cause variations in drift time (DT). DT can be converted into CCS, adding a new dimension to metabolomics. CCS is highly reproducible across instruments and labs, making it a reliable tool for compound identification. IMS is widely used in metabolomics to resolve complex mixtures and improve identification accuracy [63].

#### Data acquisition techniques

Accurate quantification of metabolites in biological samples drives MS-based metabolomics, enabling the study of metabolic changes caused by diseases and drugs. In low-resolution MS, Data acquisition modes include selected ion monitoring (SIM), selected reaction monitoring (SRM), multiple reaction monitoring (MRM), or dynamic multiple reaction monitoring (dMRM) [13, 64]. SIM, typically used with single quadrupole MS, focuses on detecting specific precursor ions [65]. This method is commonly employed in TCM efficacy studies for component identifying and quantifying key components. For example, Li et al. utilized the SIM method to quantitatively analyze 21 components in Shengmai injection [66]. Since SIM can select only one ion at a time, it requires high mass spectrometer sensitivity to maintain a sufficient signal-to-noise (S/N). MRM is considered the most reliable method for detecting hundreds of metabolites in low-resolution tandem MS. It requires selecting at least two precursor/product ion channels to obtain fragment information for target compounds. Each ion selection excludes background signals, resulting in higher resolution and sensitivity compared to SIM methods [26, 67]. For example, Yan et al. used MRM method to analyze 421 flavonoid compounds in Astragalus membranaceus, highlighting its applicability for complex compound profiling [68]. However, increasing the number of analytes (> 200) in a single scan reduces dwell time for each channel, lowering sensitivity. Alternatively, extending MS scan time per cycle decreases the data points for each chromatographic peak, compromising quantitative analysis quality [4]. Dividing chromatographic separation into time segments and scanning only around the retention times (RTs) of analytes allocates more scan time per analyte, enhancing sensitivity. This method is called dMRM, effectively detects a broader range of compounds [4]. Channel selection for MRM in metabolomics requires analyte standards, but their availability is a significant limitation of MRM [67]. In such cases, researchers have developed pseudotargeted MRM technology, which selects channels based on fragment ions from HRMS, and this approach has been successfully applied in multiple studies [62, 67].

**Fig. 4:**
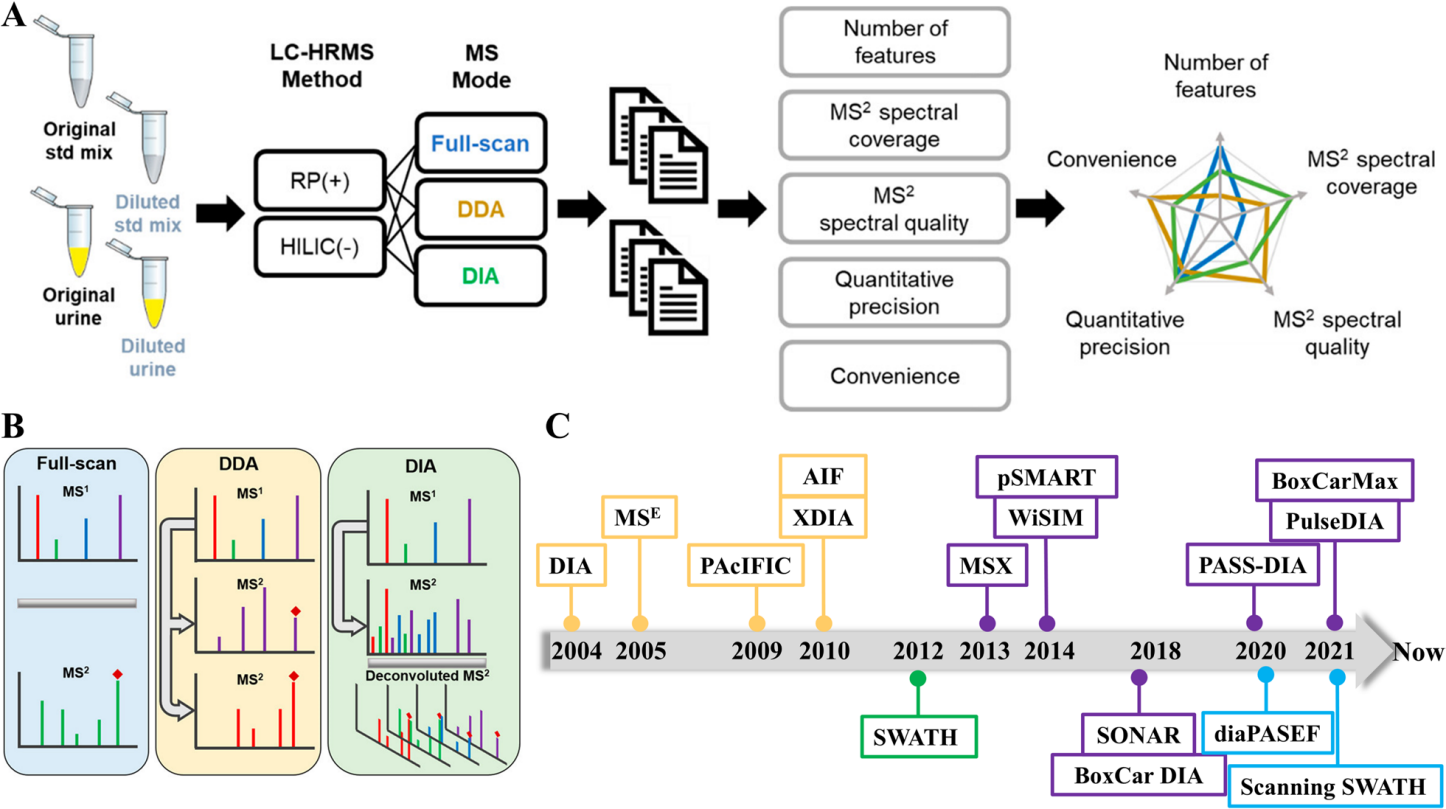
Data acquisition modes of MS. Schematic workflow **A** and the comparison **B** of full-scan, DDA, and DIA modes [69], timeline of developments of DIA approaches used in MS analysis **C**. The time for different DIA acquisition schemes, traditional method (orange), SWATH (green), SWATH-based method (purple), 4-dimension method (blue) [70–72]

Metabolomics techniques based on HRMS include GC-HRMS and LC-HRMS. In GC-HRMS, EI is the primary ionization technique. Due to its extensive fragmentation of precursor ions, data acquisition relies on full scan mode [4, 10]. In LC-HRMS predominantly employs two scanning modes: data-dependent acquisition (DDA), which focuses on selected ions, and data-independent acquisition (DIA), which collects fragment data for all ions [4, 15]. Figure 4A, B compare workflows of the three scanning modes: full scan, DDA, and DIA [69]. In DDA mode, the MS automatically switches from full scan to MS2 when precursor ions exceed intensity thresholds or match criteria such as isotope pattern, mass defect, diagnostic ion and neutral loss (NL) [4, 64]. Consequently, DDA provides clean MS2 spectra and correlates fragment ions with their parent ions. In addition, DDA methods including NL-triggered MS3 method, the mass tag-triggered DDA, and step-wise precursor ion list-based raster-mass defect filtering-triggered DDA, designed to improve MS2 data acquisition [13]. For example, Licha et al. used DDA to analyzed mice plasma after a ketogenic diet, investigating the relationship between metabolic profiles and tumor growth inhibition [73]. However, DDA struggles to detect low-abundance precursor ions, limiting its ability to capture the full metabolic profile of biological samples. Improved DDA strategies, such as iterative exclusion DDA (IE-DDA) and dpDDA, address this limitation. IE-DDA enhances metabolite detection by performing multiple injections of the same sample, targeting different precursor ions in each run [74]. dpDDA uses an inclusion list of differential and preidentified ions, combining full-scan mode on quality control samples with selective MS2 acquisition for targeted metabolite identification [75].

The DIA method analyzes fragment ions without ion-specific prior understanding of relevant ions, covering all or selected precursor ions in specific mass windows [4, 15]. Figure 4C shows the development of various DIA methods, highlighting their advancements [70, 72]. DIA encompasses several modes depending on the HRMS system: all ion fragmentation (AIF) in Orbitrap systems (Thermo Fisher Scientific), MS^E^ (elevated energy MS) and SONAR in Q-TOF system (Waters), MS^ALL^ and SWATH™ in Triple TOF system (AB SCIEX) [4]. In AIF, precursor ions are fragmented in the high-energy collision (HCD) pool, MS^E^ enriches fragment ion data with elevated higher collision energy (CE) for structural analysis, MS^ALL^ ensures broad molecular ion coverage at low CE, SONAR alternates high and low CE scans within a mass range for enhanced resolution and specificity, SWATH™ employs sequential narrow windows to fragment ions within a mass range, offering high selectivity and rapid scanning [64, 70, 72]. In summary, DIA offers high-efficiency MS2 spectra, unrestricted precursor ion selection for comprehensive compound coverage, and complete MS1 and MS2 spectra with a broad dynamic range, high sensitivity, and reproducibility, making it ideal for untargeted metabolomics analysis requiring extensive data acquisition.

#### Data processing and annotation

MS/MS spectra frequently contain fragment ions from multiple precursor ions, complicating the assignment of fragments to their precursors, especially with co-eluting compounds. Reliable deconvolution and data processing algorithms are crucial for establishing these connections [4, 15]. Unlike GC–MS, LC–MS spectra vary by instrument, requiring robust software for peak extraction and metabolite annotation. Supplementary file Table S2 lists software and database commonly used for these purposes [18, 64].

To address instrument variability, researchers have developed software based on advanced algorithms for fragment ion attribution, enabling accurate peak detection, alignment, extraction and identification. Open-source tools such as XCMS, MS DIAL, MZmine and MetaboAnalyst, as well as commercial software like Progenesis QI, UNIFI, MakerLynx, Compound Discoverer, MassHunter and Mass Profiler Pro, streamline data processing workflows [4, 6, 10, 15]. These tools integrate raw MS data processing into automated workflows, simplifying metabolite extraction from large-scale datasets. Although these programs can process hundreds of data points simultaneously, they require computers with large amounts of RAM and hard disk local space [76]. Additionally, these tools rely on external MS/MS databases for matching and identification, such as MassBank, METLIN, HMDB, NIST, LipidMaps, mzCloud, GNPS and so on [4, 10, 18, 77]. Researchers have enhanced metabolite annotation efficiency, accuracy, and coverage by developing internal MS/MS databases, such as FlavonQ, PCDL, PlantMAT, which complement externa online databases [4]. Tools such as MS-Finder, CFM-ID, MetFrag, MassFrontier, LipidBlast aid in predicting fragment information and identifying positional isomers, enhancing the annotation of unknown metabolites [4, 6, 10, 77, 78]. Standardizing chemical structure information for identified metabolites is crucial and can be facilitated by online resources including PubChem, ChemSpider, ChemicalBook, ChEBI, KEGG etc. [77, 78].

## Metabolomics technology

### Metabolomics concepts

Metabolites, as metabolic intermediates or products, reflect biological regulation in response to genetic and environmental changes, acting as crucial mediators between genotype and phenotype. Metabolomics, a field established in the late 1990 s, involves the comprehensive analysis of small molecules in organisms to elucidate their functional roles [76]. This approach has proven valuable in identifying biomarkers related to various stimuli and has applications across disciplines, including botany, nutrition, pharmaceuticals, disease diagnosis, and environmental science, enhancing our understanding of biological processes [76, 82]. In recent decades, metabolomics has advanced significantly, with MS-based metabolomics approaches becoming as the most widely used [7]. However, meaningful results require well-designed experiments tailored to specific research objectives. This involves pre-analytical steps such as sample collection and storage, the selection of suitable analytical techniques, and ensuring data quality (Fig. 2). The generated metabolic profiles are then analyzed through chemometric calculations, biological annotation, data visualization, and in-depth data mining to extract valuable insights [16]. Therefore, MS-based metabolomics integrates analytical chemistry, biochemistry, bioinformatics, and biostatistics, driving advancements in these fields. It can also be combined with other omics technologies, including proteomics, genomics, and transcriptomics, to study dynamic metabolic changes in biological systems. For instance, Pang et al. employed metabolomics and proteomics to identify changes in metabolite and proteins associated with the salvage pathway in microcephaly [83].

### Metabolomics classification

Metabolomics, the study of changes in metabolites within biological systems, has evolved with advancements in analytical techniques and increasing biomedical research demands. This field focus on diverse objectives, including broad metabolite profiling, precise quantification of metabolic pathways, single-cell analysis and dynamic metabolic flux studies. These advancements underline metabolomics’ critical role in understanding disease mechanisms, drug development, personalized medicine, and environmental science, helping researchers select appropriate methods to explore biological processes and metabolic networks [7]. Traditionally classified into targeted and untargeted approaches, metabolomics has expanded to include functional metabolomics, spatial metabolomics, SCM, and MFA (Table S3), which are discussed in the following sections.

#### Targeted and untargeted metabolomics

**Fig. 5:**
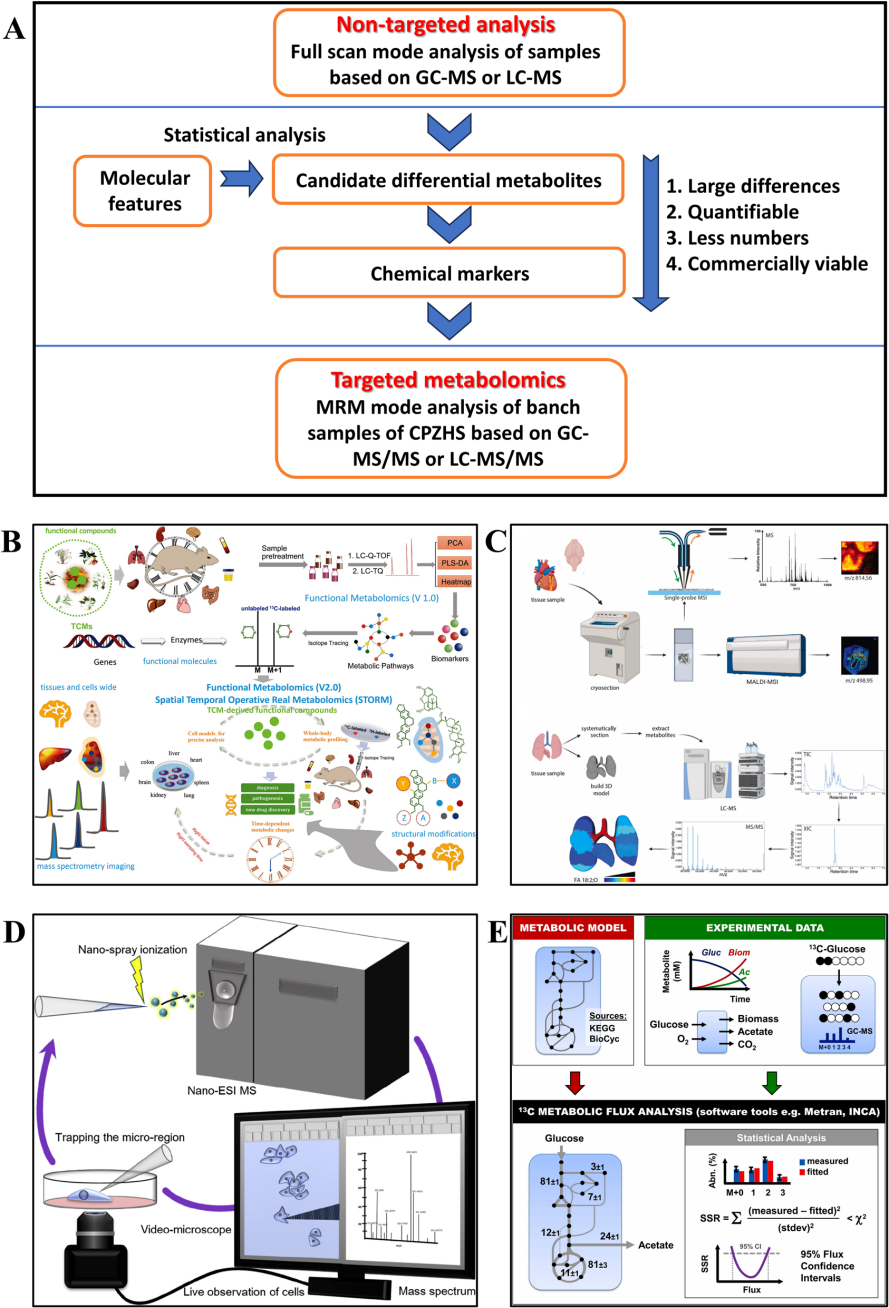
Workflows of metabolomics. **A** Non-targeted and targeted metabolomics strategy for identification of TCM [79]. **B** An updated functional metabolomics in the modern exploration of TCM derived functional compounds [3]. **C** General overview of spatial metabolomics workflow [80]. **D** Schematics of SCM [57]. **E** Integrative metabolic flux analysis of various quantitative techniques [81]

Targeted metabolomics focuses on the quantitative analysis of known metabolites, allowing researchers to study specific metabolic pathways or metabolite groups using MS, particularly MS/MS, which offers high sensitivity and accuracy (Fig. 5A) [79]. This approach is suitable for validating biological hypotheses and studying the roles of metabolites in physiological and pathological processes, with applications in identifying disease biomarkers and therapeutic targets [8]. In TCM research, targeted metabolomics has become indispensable. For example, Ding et al. used GC–MS/MS with MRM acquisition to analyze compounds in natural and artificial musk, achieving traceability in TCM [79]. However, this approach is limited to predefined metabolites, offering lower coverage and partial information on the sample’s overall composition (Table S3).

Untargeted metabolomics is an exploratory approach that aims to comprehensively profile metabolites in biological samples using HRMS such as TOF, Orbitrap, and IMS. This method detects a wide range of metabolites, followed by identification and quantification through data analysis, facilitating the discovery of new biomarkers and the elucidation of previously unknown metabolic pathways [84]. It is widely applied in TCM research to analyze complex metabolic profiles. For example, Ye et al. used UPLC-Q-TOF/MS to analyze urine and blood metabolomes in gastroesophageal reflux disease, identifying three metabolites as biomarkers for TCM syndrome classification [85]. While untargeted metabolomics offers high-throughput detection and broader metabolite coverage, it has lower accuracy compared to targeted approaches (Table S3). Therefore, integrating targeted and untargeted metabolomics is often necessary to achieve both high precision and comprehensive coverage.

#### Functional metabolomics

Functional metabolomics integrates metabolomics with functional assays to explore biological roles and mechanisms of specific metabolites, extending beyond detection and quantification to explore their functions in cellular signaling pathways (Table S3 and Fig. 5B) [3]. This emerging, application-oriented field emphasizes integrating metabolite biology into broader omics research, making it particularly relevant to interdisciplinary areas such as molecular biology, cell biology, and biochemistry [86]. In TCM research, functional metabolomics has been instrumental in uncovering the mechanisms underlying therapeutic efficacy. For example, Jing et al. discovered that berberine treats dextran sulfate sodium-induced colitis in rats by activating AhR through microbial tryptophan catabolites [87]. These studies highlight how functional metabolomics identifies and elucidates the active compounds driving TCM’s therapeutic effects, providing a scientific foundation for understanding how these components modulate disease-related biomarkers [88].

#### Spatial metabolomics

The production, accumulation, and consumption of metabolites are closely tied to their spatiotemporal distribution in cells, tissues, organs, making the study of this heterogeneity essential for understanding complex physiological and pathological processes [89]. Similarly, the interactions between active compounds and disease targets also show distinct spatiotemporal patterns. In this context, spatial metabolomics has emerged as a key field, enabling the localization and in situ profiling of metabolites within biological systems (Table S3 and Fig. 5C) [80]. Primarily achieved by MS imaging (MSI), spatial metabolomics offers label-free, non-specific detection with broad metabolite coverage and visualization capabilities [7, 89]. This approach has proven valuable for studying the biosynthesis and transport pathways of endogenous metabolites in animals and plants, as well as the ADME of exogenous drugs, aiding drug development. For example, Meng et al. used MSI to investigate the lysosomal release and nuclear entry of anticancer drugs from nanoparticle carriers, revealing drug delivery mechanisms [90]. Additionally, they mapped metabolite distribution in parsnip roots with MSI, providing insights into plant metabolic pathways [91].

#### Single-cell metabolomics (SCM)

Cellular heterogeneity, influenced by genetic, epigenetic, and environmental factors, pervasive biology, influencing cell morphology, physiology, and pathology [92]. Conventional metabolomics provides holistic results, overlooking intercellular variations and failing to capture the true metabolic state of individual cells [93]. Single-cell omics, including SCM, have evolved significantly, encompassing genomics, transcriptomics, proteomics, and more. Metabolites, as links between genes and phenotypes, have driven the emergence of SCM [94]. SCM is a high-sensitivity technique that analyzes metabolites within individual cells, allowing the characterization of dynamic metabolic state at the cellular level (Fig. 5D) [57, 95]. Compared to traditional metabolomics, SCM reveals metabolic heterogeneity between cells, critical for understanding individual differences in complex biological systems (Table S3). In TCM research, SCM quantifies the spatial distribution of drugs and metabolites, aiding research on pharmacokinetics, target identification, and therapeutic mechanisms [96]. However, SCM application in TCM efficacy research remain limited, with most studies focusing on pharmaceutical drug development, illustrating differential drug effects across cell types [93, 97].

#### Metabolic flux analysis (MFA)

Metabolism in living organisms is a dynamic biochemical reaction network, with metabolite concentrations and pathway activities reflecting the overall state of biological system through dynamic accumulation and consumption rates [7, 98]. Based on this principle, MFA emerged to explore pathway activities, quantified as the flux of substances per unit of time [99]. By integrating metabolite concentrations and fluxes, MFA combined with conventional metabolomics provides comprehensive insights into metabolic regulatory networks and biological mechanisms (Fig. 5E and Table S3) [81, 98]. However, flux cannot be directly measured by MS. Thus, MFA utilizes isotope tracers to track the enrichment rate and metabolic fate of downstream metabolites. The choice of tracer and labeling pattern depends on the specific metabolic flux under investigation [86, 99]. Detailed analytical methods for MFA are extensively discussed in other references [99–101]. MFA has found broad applications in the biomedicine, particularly for identification therapeutic targets in cancer treatment. It has been used to explore metabolic pathways such as glycolysis, the tricarboxylic acid cycle, glutamine metabolism, and lipid metabolism [100, 101]. In pharmaceuticals, MFA primarily investigates the mechanisms and resistance of chemical drugs. However, its application in TCM remains limited, highlighting the need for further exploration [100].

### Metabolomics analysis strategy

#### Study design

To maintain the integrity and precision of experimental outcomes, researchers need to clearly articulate their hypotheses, research questions, and goals before starting experiments. This clarity ensures that the experimental design matches the data needs for the biological inquiries at hand. In metabolomics, close collaboration with sample collection teams is essential for proper experimental groupings. Researchers must consider instrument capabilities, sample stability, and availability, selecting the appropriate analytical methods for their samples before conducting experiments. Ethical considerations and animal welfare must also be adhered to in accordance with relevant regulations [15, 18].

In metabolomics, optimizing sample size, collection, preprocessing, analytical methods, and data processing is crucial for reducing statistical noise and ensuring data quality [15]. For TCM efficacy analysis, raw data are influenced by preparation methods, plant species, parts, and environmental conditions, as well as the growth conditions of model organisms in vivo, culture conditions of cells in vitro, and sampling methods for ex vivo tissues [102]. Longitudinal cohort studies are vital for assessing TCM efficacy clinically [16]. Metabolomics investigates metabolites, pathways, and biomarkers related to TCM efficacy mechanisms, requiring reliable chemometric methods and datasets, with data volume dependent on sample size [16]. Currently, this field lacks standardized experimental designs, necessitating customized approaches based on specific biological questions, while accounting for sample and resource limitations.

#### Chemometrics

MS-based metabolomics generates extensive data that necessitates sophisticated statistical analysis, known as chemometrics, for extracting biological insights. This section, following Sect."Data processing and annotation"discussion on raw data and peak annotation, focuses on interpreting and correlating identified metabolites across sample groups. Table S4 summarizes common statistical methods used in this context. Adequate sample size is essential for statistical analysis, especially in rare disease studies where samples are limited. Small samples and large datasets can lead to spurious correlations and misinterpretation of potential biomarkers [15]. Univariate and multivariate statistical analyses are commonly used; univariate analysis, for instance, examines metabolite abundance changes using t-tests and analysis of variance (ANOVA) [103]. Results are often displayed as means or medians in box or violin plots, with significance levels indicated. For large datasets, Bonferroni correction is used to control the error rate and minimize false positives [104]. Multivariate analysis includes unsupervised and supervised methods (Table S4) [105]. Unsupervised methods use clustering or dimensionality reduction algorithms to identify trends and differentiate samples within datasets [106]. In contrast, supervised methods perform discriminant analysis on predefined datasets, often leveraging machine learning and deep learning techniques [107]. The specific principles and applications of various algorithms are beyond the scope of this review, and readers may refer to other studies for further information [18].

Unsupervised models are typically used to identify chemical fingerprints in TCM [102]. For instance, Zhu et al. used principal component analysis (PCA) and hierarchical cluster analysis (HCA) to analyze 14 indicators and explore the chemical fingerprints of different Fuling Decoction levels [108]. In bioactivity studies, these chemometric tools are often used to model relationship between chemical fingerprints and bioactivity. For example, Shawky et al. used statistical tools to correlate between chemical fingerprints with efficacy and applied cluster analysis to identify potential biomarkers in N. sativa oils with antibacterial, anti-inflammatory, and analgesic effects [109].

#### Biological function annotation

After mining the data matrix with chemometric methods, metabolic pathway analysis and biological function annotation must be performed on selected metabolites. Pathway analysis links experimental phenotypes to altered metabolic pathways, using tools like MetaCyc, KEGG, LipidMaps, MetaboAnalyst (Table S2). However, challenges in obtaining comprehensive metabolite information often complicate the interpretation of their biological functions. Network analysis integrates metabolites into biochemical networks to clarify their roles in metabolic pathways, with tools such as ReconMap and MetExplore facilitating this process [110, 111]. The complexity of TCM, characterized by multi-component, multi-target, and multi-pathway interactions, has led to the development of network pharmacology. This approach examines the relationships among chemical fingerprints, metabolic fingerprints, and network targets, highlighting TCM’s holistic and systemic effects. It involves steps like collecting TCM chemical components, predicting their targets using protein databases, and constructing component-target-disease networks. This methodology is critical for understanding TCM efficacy, targets, and toxicity [112]. Network pharmacology, often combined with machine learning, aids in identifying active TCM components, evaluating efficacy, and elucidating pharmacological mechanisms. Integrating metabolomics with network pharmacology provides a powerful framework for studying interactions between TCM components and organisms. For instance, Zhou et al. explored compound-target-disease relationships in Chuanxiong Rhizoma-Xiangfu Rhizoma [113], while Yu et al. identified three endogenous biomarkers and six exogenous quality biomarkers through metabolomics and network pharmacology analysis [114]. Finally, metabolites interact with genes and proteins to induce phenotypic and functional changes, forming just one aspect of biochemical reactions. Therefore, annotating their biological functions requires integrating metabolomics with other omics, such as genomics, transcriptomics, microbiomics, and proteomics, to better understand metabolic networks, biological processes, and mechanisms [6, 8].

## Metabolomic analysis under different conditions in TCMs

Unlike the “one-size-fits-all” approach of Western medicine, TCM focuses on holistic regulation of the body’s metabolic activity and serves as a crucial source for drug discovery, gaining global attention. In the USA, 25% of nature medicines approved by the Food and Drug Administration (FDA) are plant-derived [115]. However, the complexity of TCM components remains a major obstacle to its development as new drugs. Metabolomic analysis, which studies endogenous small molecule metabolites in plants and animals, is increasingly used in drug development. It is also a powerful tool for evaluating TCM efficacy and pharmacological mechanisms. MS-based metabolomic methods are particularly effective in analyzing TCM composition, elucidating mechanisms, identifying targets, and exploring disease pathways, greatly advancing TCM efficacy research [7].

### Characterization of chemical components

**Fig. 6:**
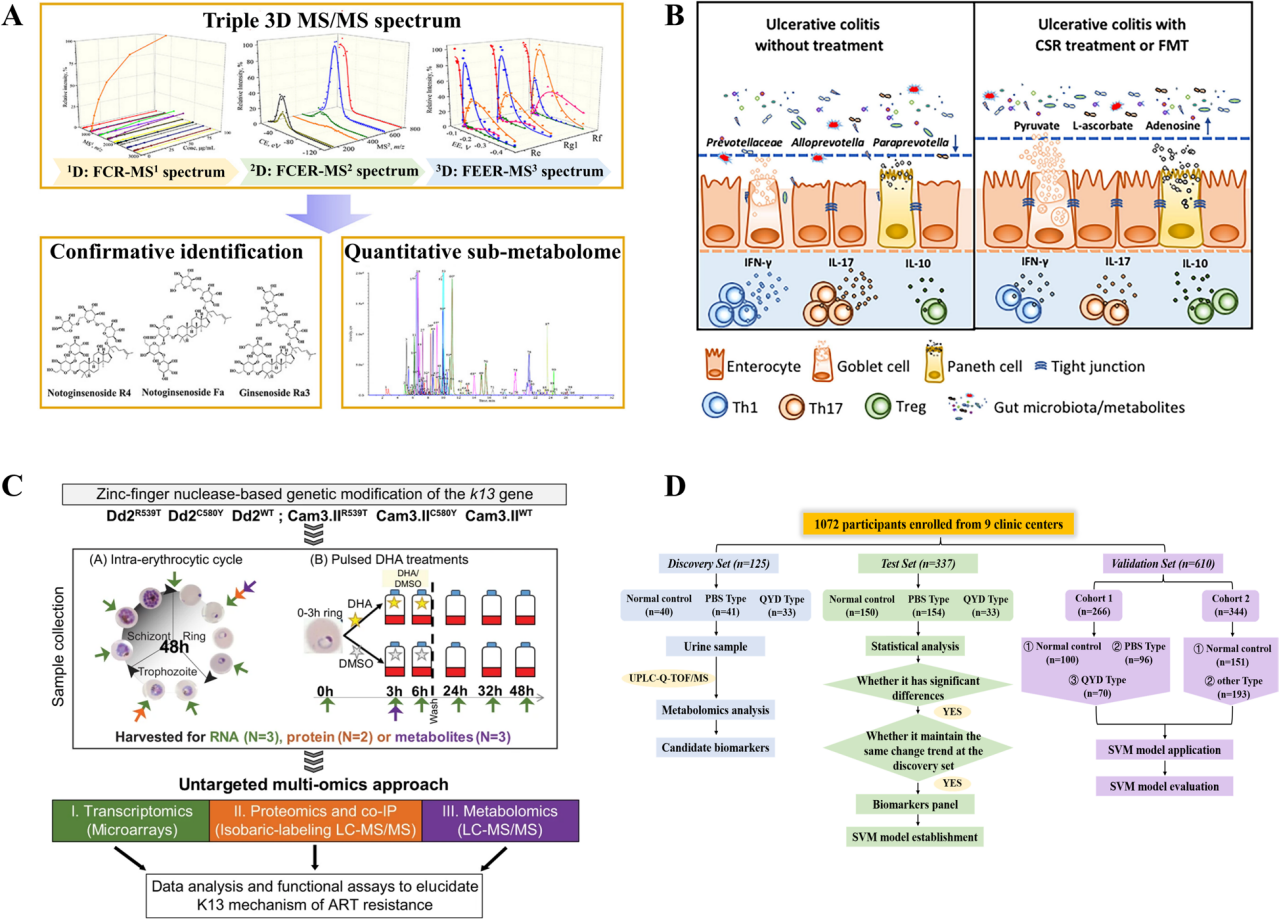
MS-based metabolomic applications under different conditions in TCM. **A** Using a tandem multi-stage MS platform and a “top-down” strategy comprehensively characterize the saponin compounds in Panax notoginseng [116]. **B** Therapeutic effects, the potential target and mechanism of CSR on treating ulcerative colitis using untargeted metabolomics, histological staining, intestinal permeability assay et al. [117]. **C** By applying metabolomics, transcriptomics, and proteomics to study the mutant K13 protect Plasmodium falciparum against the effects of artemisinin [118]. **D** Using MS-based metabolomics technology, researchers aim to identify biomarkers for two syndromes (phlegm-stasis syndrome and qi-yin deficiency syndrome) in coronary heart disease patients [119]

Identifying components in TCM is a crucial first step in efficacy research. TCM’s endogenous components vary with preparation methods, plant species, plant parts, and growing conditions [102]. Despite this variability, the complex of TCM samples can be addressed using MS-based metabolomics, which combined with advanced analytical tools and efficient data processing, allows for simultaneous qualitative and quantitative analysis of thousands of metabolites. Numerous studies have used MS to characterize TCM’s chemical composition. For example, Xia et al. used LC-QTOF with MS^All^ and SWATH data acquisition methods to identify 89 triterpene saponins in Acanthopanax senticosus, including the first reported occurrence of malonylsaponin in this TCM [120]. Previously discussed separation and MS techniques provide a foundation for analyzing TCM components. Researchers can design analytical strategies for characterizing TCM components based on laboratory conditions and research objectives. For example, tandem multi-stage MS, combined with untargeted and targeted metabolomics, has been used to precisely characterize and quantify the structurally diverse saponin compounds in *Panax notoginseng*, which include numerous isomers. This “top-down” strategy offers a comprehensive method for studying saponins in *Panax notoginseng* (Fig. 6A) [116]. In addition, MS-based metabolomics technology has also proven effective in identifying TCM’s key active substances. Traditional methods involve separating crude extracts and testing for bioactivity, but it’s time-consuming and inefficient. Metabolomics, focusing on small molecule metabolites, has become a vital tool for TCM drug discovery and post-approval drug monitoring [121]. By using chemometric analysis to screen bioactive compounds, metabolomics has successfully identified active TCM components such as kaempferol, betulinic acid, and geniposide [115].

### Target identification

In 1996, the Chinese government issued the “Action Plan for the Modernization of Traditional Chinese Medicine Science and Technology Industry”. After 30 years of exploration, researchers have isolated and characterized more 6,000 natural products from TCMs, including numerous active components with potential for new drug development [122]. However, the therapeutic effects of these active components and their molecular targets remain largely unexplored. Identifying the molecular targets of active components is key to understanding drug mechanisms in disease models and advancing clinical research and personalized medicine. In recent years, growing evidence highlights the critical role of metabolomics in identifying molecular targets for TCMs. Researchers have utilized MS-based metabolomics in conjunction with multi-omics techniques to discover that Celastrol exert anti-inflammatory effects by modulating Treg/TH1 and Treg/Th17 balances, blocking the NF-κB pathway, reducing pro-inflammatory cytokines (IL-6, TNF-α, and IL-1β), and modulating microbiota structure and the metabolome (Fig. 6B) [117]. Furthermore, Celastrol has also been shown to suppress vascular calcification by inhibiting BMPRII and NOX2 expression, activating Smad6 and HO-1, and blocking the BMP2/Smad1/5 and Wnt/β-catenin signaling pathways [123]. Disease biomarkers serve as intermediaries between the active components of TCM and their therapeutic effects. Monitoring the metabolic status of these markers allows researchers to assess the availability of active components and elucidate biomarkers related metabolic processes in critical disease pathways targeted by drugs.

### Mechanisms of action

Insufficient understanding of the MoA of TCM extracts or active components remains a major obstacle to the modernization of TCM due to their complexity and lack of clarity. Researchers use chemometrics and bioinformatics to explore the MoA of active TCM ingredients, offering scientific insights into TCM quality standards [124]. Artemisinin, an active ingredient extracted from the aerial parts of Artemisia annua, has potential for treating malaria, cancer, and lupus erythematosus [125]. Further studies on artemisinin have revealed that Atovaquone reverses artemisinin resistance by modulating mitochondrial metabolism. Specifically, the K13 mutation alters mitochondrial metabolism in Plasmodium falciparum, with non-targeted metabolomics identifying changes in metabolites involved in the TCA cycle, purine, glutamate, and pyruvate metabolism, further promoting artemisinin resistance (Fig. 6C) [118]. Ginseng, a widely studied and highly valued TCM material, contains ginsenosides as its primary active components. These are absorbed into the bloodstream and metabolized into rare saponins through intestinal microbiota de-glycosylation, contributing to various biological functions [126]. Lin et al. used non-targeted metabolomics to analyze serum and culture media from obese mice treated with ginseng extract, discovering that it induces Enterococcus faecalis to produce the unsaturated fatty acid myristic acid. This fatty acid activates brown adipose tissue and promotes beige fat formation, reducing obesity incidence and providing key evidence for the therapeutic potential of ginseng extract in metabolic diseases [127].

### TCM syndrome

Unlike the personalized treatment of Western medicine, TCM emphasizes syndrome differentiation and treatment. Disease-related physiological changes manifest as corresponding syndromes, which are reflected in endogenous metabolites in various disease models (Fig. 1). Therefore, characterizing endogenous metabolite changes associated with TCM syndromes is essential for clarifying the mechanisms underlying TCM treatments. Extensive research has demonstrated that metabolomics is a powerful tool for comprehensively depicting TCM syndromes and distinguishing patients with different symptoms to enable syndrome differentiation and treatment. Applications in this area mainly focus on the diagnosis and intervention of TCM syndromes. Zhou et al., utilizing metabolomics, identified 15 biomarkers associated with phlegm and blood stasis syndrome and 12 biomarkers linked to qi and yin deficiency syndrome in the urine samples of 1072 patients with coronary heart disease (CHD). Using the SVM prediction model, they demonstrated that these markers could predict these two syndromes in CHD with an accuracy rate of 98.0% (Fig. 6D) [119]. Similarly, Jiang et al. identified differential metabolites in diabetic patients with “Kidney-Yin Deficiency Syndrome” linked to amino acid metabolism, energy metabolism, and gut microbiota changes [128]. Therefore, metabolomics is a valuable tool for studying the principles of TCM syndrome theory and its therapeutic mechanisms.

## Summary and future perspectives

### Future perspectives

AI and emerging technologies offer significant potential to advance TCM efficacy research, particularly when integrated with MS-based metabolomics [129]. While metabolomics provides precise identification of complex TCM components and active metabolites, its high costs and operational complexity limit broader applications in research and clinical practice. AI and big data technologies help address these challenges by creating open data platforms, reducing costs, and improving efficiency and accuracy.

AI-driven methods, such as computational deconvolution and structure-to-activity prediction, accelerate the structural and functional analysis of TCM metabolites [130]. For instance, deep learning architectures (e.g., Metabolite Inference with Spectrum Transformers) innovates by integrating a “chemical formula transformer” to process tandem MS spectra, aiming to bridge the substantial knowledge gap in untargeted MS studies [131]. Integrative algorithms like CSI:FingerID further reduce false positives by synergizing multi-parametric data (m/z, RT, CCS) with graph-based structure prediction, enabling reliable identification of isomeric flavonoids and saponins [132]. These advances address the “unknowns” bottleneck in TCM metabolomics. When combined with network pharmacology, AI can reveal mechanisms of action, identify therapeutic targets, and navigate complex interactions, supporting clinical efficacy validation and promoting TCM standardization and global integration [133]. Bai et al. presents a DrugBAN, deep bilinear attention network (BAN) framework with domain adaptation to explicitly learn pairwise local interactions between drugs and targets, with conditional domain adversarial learning to align learned interaction representations across different distributions for better generalization on novel drug–target pairs [134]. Additionally, emerging technologies such as organoids, 3D printing, and microchip systems, combined with TCM theory and other disciplines, provide innovative tools to explore the deeper mechanisms of TCM [135]. Despite these advances, the opaque nature of AI algorithms and the risk of false positives require rigorous validation to ensure reliability and practical application. By addressing these limitations and fostering interdisciplinary collaboration, AI and new technologies can transform TCM research, enhancing its accessibility, accuracy, and global impact.

The integration of metabolomics into TCM research holds transformative potential for bridging traditional knowledge and modern drug development. To realize this vision, three strategic directions warrant emphasis: (1) accelerating TCM clinical trials through metabolite-based phenotyping and real-time biomarker monitoring; (2) aligning with global regulatory frameworks via adherence to Food and Drug Administration (FDA) and European Medicines Agency (EMA) guidelines on botanical drugs and leveraging standardized reference materials like NIST SRM 1950; and (3) fostering international collaboration through initiatives such as the EMA-National Medical Products Administration (NMPA) partnership and the FDA-NIH Biomarker Consortium. Specifically, metabolite profiling (e.g., serum bile acid analysis) enhances patient stratification, while LC–MS/MS-driven monitoring enables early detection of toxicity markers, guiding safer dosing. Regulatory compliance is strengthened by public data repositories (e.g., MetaboLights) and inter-lab calibration protocols. Collaborative efforts validate TCM-specific biomarkers and streamline multi-regional approvals, positioning metabolomics as a cornerstone of TCM modernization.

### Future challenges

Metabolomics, as a relatively new field, faces significant challenges despite progress in standardizing sample preparation, data acquisition, and qualitative analysis. A key issue is the reliance on experimental expertise for metabolite annotation, leading to subjective identification outcomes [129]. Developing standardized tools and methods through AI and bioinformatics is essential to reduce bias and improve accuracy in metabolite identification.

Another major challenge is the limited size of MS databases, which hinder identification efficiency. Incomplete fragmentation due to insufficient CID energy further exacerbates this issue. Expanding MS compound databases and leveraging AI-driven machine learning to predict fragmentation patterns are critical steps to enhance the identification of metabolites, particularly in complex TCM systems. Moreover, Bioinformatics databases like KEGG, HMDB, TCMSP, and TCMID are invaluable for TCM research but often suffer from outdated entries and data gaps, resulting in inconsistencies. Regular updates and refinements are crucial to ensure accurate and reliable data support for metabolomics and TCM efficacy studies. Therefore, standardizing data processing and expanding metabolomics databases are fundamental to advancing MS-based metabolomics and its application in TCM, addressing current limitations and paving the way for more robust and accurate research. For instance, tackling metabolomics challenges requires globally coordinated efforts. International alliances like METLIN-SMRT have built open-access compound libraries through federated data sharing [136], while AI-driven tools (MS-DIAL, QI) enable reproducible data preprocessing without manual intervention (Sect."Data processing and annotation").

## Supplementary Information

Supplementary Material 1

## Data Availability

Not applicable.

## Abbreviations

MS: Mass spectrometry
TCM: Traditional Chinese medicine
AI: Artificial intelligence
MoA: Mechanisms of action
ADME: Absorption, distribution, metabolism, and excretion
HRMS: High-resolution mass spectrometry
GC: Gas chromatography
LC: Liquid chromatography
CCS: Collision cross sections
LC: Liquid chromatographic
GC: Gas chromatographic
SLE: Solid–liquid extraction
LLE: Liquid–liquid extraction
MDC: Multidimensional chromatography
HPLC: High-performance liquid chromatography
UHPLC: Ultra-high-performance liquid chromatography
RPLC: Reverse phase liquid chromatography
HILIC: Hydrophilic interaction chromatography
NP: Normal phase
^1^D: One-dimensional
^2^D: Two-dimensional
EI: Electron ionization
NIST: National institute of standards and technology
ESI: Electrospray ionization
ME: Matrix effect
nano-ESI: Nano-electrospray ionization
InESI: Induced nano-electrospray ionization
ESSI: Electrosonic spray ionization
EESI: Extractive electrospray ionization
DESI: Desorption electrospray ionization
MALDI: Matrix-assisted laser desorption/ionization
APCI: Atmospheric pressure chemical ionization
APPI: Atmospheric pressure photoionization
DART-MS: Direct analysis in real-time mass spectrometry
QQQ: Triple quadrupole
IT: Ion trap
TOF: Time-of-flight
FT-ICR: Fourier transform ion cyclotron resonance
IMS: Ion mobility spectrometry
DT: Drift time
SIM: Selected ion monitoring
SRM: Selected reaction monitoring
MRM: Multiple reaction monitoring
dMRM: Dynamic multiple reaction monitoring
RT: Retention time
S/N: Signal-to-noise
DDA: Data-dependent acquisition
DIA: Data-independent acquisition
NL: Neutral loss
IE-DDA: Iterative exclusion DDA
dpDDA: Differential and preidentified ions DDA
AIF: All ion fragmentation
HCD: High-energy collision
CE: Collision energy
MSI: MS imaging
SCM: Single-cell metabolomics
MFA: Metabolic flux analysis
ANOVA: Analysis of variance
PCA: Principal component analysis
HCA: Hierarchical cluster analysis
FDA: Food and drug administration
EMA: European medicines agency
NMPA: National medical products administration
BAN: Bilinear attention network
