# Supplementary material for: Mass spectrometry-based metabolomic as a powerful tool to unravel the component and mechanism in TCM
Source: Chin Med. 2025 May 12;20:62. doi: 10.1186/s13020-025-01112-2 (PMC12067679; doi:10.1186/s13020-025-01112-2)
Supplement: Supplementary file 1 — Supplementary Material 1 [file 13020_2025_1112_MOESM1_ESM.docx]

Supporting information

**Mass spectrometry-based metabolomic as a powerful tool to unravel the component and mechanism in TCM**

Guang-Qin Liao^a,b^, Hong-Mei Tang^a,c^, Yuan-Di Yu^b,c^, Li-Zhi Fu^a,d^, Shuang-Jiao Li^e^, Mai-Xun Zhu^a,b,*^

^a^ Chongqing Academy of Animal Sciences, Chongqing 402460, China

^b^ National Center of Technology Innovation for Pigs, Chongqing 402460, China

^c^ National Animal Disease-Chongqing Monitoring Station, Chongqing 402460, China

^d^ Chongqing Research Center of Veterinary Biologicals engineering and technology, Chongqing 402460, China

^e^ Chinese Academy of Agricultural Sciences, Beijing 100061, China

^*^ Corresponding author

E-mail addresses: [lgqlightcheng@163.com](mailto:lgqlightcheng@163.com) (Guang-Qin Liao), [tanghongmei2005@126.com](mailto:tanghongmei2005@126.com) (Hong-Mei Tang), [yuyd@cqaa.cn](mailto:yuyd@cqaa.cn) (Yuan-Di Yu), [fulz@cqaa.cn](mailto:fulz@cqaa.cn) (Li-Zhi Fu), [lsj0926@yeah.net](mailto:lsj0926@yeah.net) (Shuang-Jiao Li), [zhumx@cqaa.cn](mailto:zhumx@cqaa.cn) (Mai-Xun Zhu)

**Table S1**

A brief comparison and selection of different MS-based [25, 64, 70].

| **Technology** | **Advantages** | | | **Disadvantages** | | |
| --- | --- | --- | --- | --- | --- | --- |
| GC-MS | Mature technology; Lower instrument maintenance  Excellent separation reproducibility  Suitable for the detection of volatile metabolites (e.g., alcohols)  Standardized EI spectra (NIST library) simplify annotation | | | Derivatization reagents increase per-sample cost Limited for polar/large molecules detection  Unable to produce parent ions  Novel compound identification is difficult  Relatively small detection range | | |
| LC-MS | Excellent sensitivity; Simple sample pretreatment process  Wide coverage of metabolite detection (e.g., amino acids)  Relatively short time for sample analysis with sub-2 μm stationary phase particles | | | ESI efficiency varies by analyte Higher solvent & column costs  Lower reproducibility than GC-MS  Complex due to variable adducts, in-source fragmentation, and matrix effects | | |
| IMS (ion mobility spectrometry) | Gold standard CCS values  Matching with multiple MS detectors  In situ detection  Providing location information of metabolites | | | Novel compound identification is difficult  Ion suppression  Imaging quality is affected by resolution  Relatively long time for sample analysis | | |
| **Instrument** | **Vendor** | **Mass accuracy ^*^** | **Mass range** | **Resolution** | **Acquisition speed** | **Dynamic** |
| **Orbitrap** |  | | | | | |
| Q-Exactive HF-X | Thermo Fisher Scientific | Relatively long time for sample analysis | 50 to 6,000 m/z | 240,000 at m/z 200 | 40 Hz | 10^3^ |
| Q-Exactive Focus | Thermo Fisher Scientific | <1 ppm | 50 to 3,000 m/z | 70,000 at m/z 200 | 12 Hz | 10^3^ |
| Orbitrap Exploris 480 | Thermo Fisher Scientific | <1 ppm | 40 to 8,000 m/z | 480,000 at m/z 200 | 40 Hz | 10^3^ |
| Orbitrap Eclipse Tribid | Thermo Fisher Scientific | <1 ppm | 50 to 8,000 m/z | 1,000,000 at m/z 200 | 40 Hz | 10^3^ |
| Orbitrap Fusion Lumos | Thermo Scientific | <1 ppm |  | 500,000 at m/z 200 | 20 Hz | 10^3^ |
| **Q-TOF** |  | | | | | |
| TripleTOF 6600 | Sciex | <1 ppm | 5 to 4,000 m/z | 35,000 at m/z 956 | 100 Hz | 10^5^ |
| timsTOF | Bruker | <0.8 ppm |  | 50,000 at m/z 1222 | >100 Hz | 10^5^ |
| 6560 Q-TOF | Agilent | <1 ppm | 100 to 10,000 m/z | 40,000 at m/z 2722 | 30 Hz | 10^5^ |
| Xevoo G2-XS | Waters | <1 ppm | 20 to 16,000 m/z | 40,000 at m/z 956 | 30 Hz | 10^4^ |
| Synapt G2-Si HDMS Q-TOF | Waters | <1 ppm | 20 to 32,000 m/z | 60,000 at m/z 956 | 30 Hz | 10^4^ |

Note: 1. Data were primarily obtained from various instrument providers' websites, potentially resulting in slight variations. The acquisition rates may differ between MS and MS/MS modes.

2. * Mass accuracy is based on internal calibration.

**Table S2**

Some of the most popular software or databases used for the annotation of features issued from MS data analyses [16, 18, 54, 77, 107].

| **MS data processing** | **Purchase** | **Type of data** | **Alignment** | **Short description** | |
| --- | --- | --- | --- | --- | --- |
| XCMS | Open source | LC-MS | RT alignment | For LC-MS data processing, featuring peak detection, alignment, and quantitation. | |
| MS DIAL | Open source | GC-MS, LC-MS | RT alignment, MS/MS spectra alignment | For MS data processing with advanced deconvolution capabilities | |
| MZmine | Open source | LC-MS | RT alignment | With tools for visualization, peak detection, and alignment. | |
| MetaboAnalyst | Open source | LC-MS | Data normalization and transformation | For metabolomic data analysis, including statistical analysis and pathway mapping. | |
| Progenesis QI | Waters | LC-MS | RT alignment, isotopic pattern matching | Known for its accuracy in quantitation and ability to handle complex datasets. | |
| UNIFI | Waters | LC-MS, UPLC-MS | RT alignment, mass accuracy calibration | Specifically designed for use with Waters instruments. | |
| MakerLynx | Waters | LC-MS | RT alignment, peak integration | For chromatographic and spectral data analysis, used mainly for high-throughput screening and quantification. | |
| Compound Discoverer | Thermo Fisher Scientific | LC-MS, Orbitrap-MS | RT alignment, mass recalibration, isotope pattern matching | For small molecule analysis, offering advanced capabilities for unknown identification, structural elucidation, and pathway mapping. | |
| MassHunter | Agilent technologies | LC-MS, GC-MS | Peak finding and deconvolution | It allows a thorough study of acquired chromatograms, extraction, and deconvolution. Compound identification based on spectra and/or RT using NIST DB and customized target libraries. | |
| Mass Profiler Professional | Agilent technologies | LC-MS, GC-MS | RT alignment, normalization, differential analysis | Analysis of complex MS data, providing statistical analysis, pathway analysis, and biomarker discovery capabilities. | |
| **Databases** | **Link** | **Pathways/Reactions** | **MS/MS** | **Compounds** | **Short description** |
| MassBank | https://massbank.eu |  | 120,184 | 16,278 | Longest standing community database |
| METLIN | https://metlin.scripps.edu |  | 431,000 | 900,000 | Developed for QTOF instruments |
| HMDB | https://hmdb.ca | 2,715/7,879 | 9,445,375 | 220,945 | Human metabolites database |
| NIST | https://chemdata.nist.gov |  | 1,390,000 | 51,501 | Curated DB, graphical interface |
| LipidMaps | <https://lipidmaps.org> | 45 | 59,258 | 48,601 | Lipidomics database |
| mzCloud | https://www.mzcloud.org/ |  | 16,531,567 | 32,330 | Multiple stage MSn |
| GNPS | https://gnps.ucsd.edu |  | 221,083 | 18,163 | Mass spectral collaborative database |
| PubChem | https://pubchem.ncbi.nlm.nih.gov |  |  | 118,576,038 | Small molecules, metadata |
| ChemSpider | https://legacy.chemspider.com/ |  |  | 180,190,612 | Small molecules, curated data |
| ChemicalBook | https://www.chemicalbook.com/ |  |  | 16,207,552 | Chemical information data |
| ChEBI | http://www.ebi.ac.uk/chebi/ |  |  | 153,614 | Molecules of biological interest |
| KEGG | https://www.kegg.jp | 7,717/24,433 |  | 27,429 | Pathway database |
| MetaCyc | https://metacyc.org | 3,153/19,020 |  | 19,372 | Pathway database, multiple species |
| Recon3D | [http://vmh.life](http://vmh.life/) | 19,313 |  | 5,607 | Three-dimensional metabolite and protein structure data |
| **In-silico prediction** | **Fragmentation Method** | **Compound Databases** | **Type of Interface** | **Short description** | |
| MS-Finder | Rule-based (hydrogen rearrangement rules) | Databases plus MINE and PubChem | Windows GUI | Inference analysis and structure identification of MS fragmentation data. | |
| CFM-ID | Hybrid rule-based machine learning | KEGG, HMDB | Web application, command line tool | Hybrid rule-based machine learning algorithm used in this tool is a probabilistic generative model for the CID fragmentation process which is learned from experimental data. | |
| MetFrag | Bond disconnection algorithm | HMDB, KEGG, PubChem | Web application, command line tool | Inferring candidates through fragment matching. | |
| MassFrontier | Rule-based (literature reaction mechanisms) | Internal MS database | Windows GUI | This tool relies on generic MS rules as well as published  fragmentation reactions, reported rearrangements, and  neutral losses. | |
| MassFragment | Bond disconnection  algorithm | Internal MS  database | Internal MS  database | This tool employs a series of chemically intelligent algorithms is based on the precursor structural system bond breaking to automatically identify product ion fragments. | |
| FingerID | Fragmentation trees and support vector machine learning | PubChem and multiple bio databases | GUI, command line tool | This tool predicts the molecular fingerprints for each query spectrum by a large set of spectra in MassBank using multiple kernel learning. | |
| MetFID | Artificial neural network |  | Command line  tool | Applies an artificial neural network with two hidden layers to predict a composite vector comprising of 528 binary entries. | |
| MAGMa | Rule-based fragmentation | PubChem, KEGG,  HMDB | Web application, command line tool | This tool excels in processing spectral trees obtained from MSn, providing a visual representation of the successive fragmentation processes. | |
| Met ISIS | Artificial neural network |  | Command line  tool | The algorithm in this tool is to perform in silico fragmentation of metabolites to find accurate bond cleavage rates employing CID-MS/MS. | |
| LipidBlast | Heuristic approaches | Internal MS database | Windows GUI, command line tool | For specific compound classes (lipids). | |

**Table S3**

Advantages and disadvantages of application of frontier technologies in metabolomics [7, 98, 94].

| **Name** | **Advantages** | **Disadvantages** |
| --- | --- | --- |
| Targeted metabolomics | It has high sensitivity and specificity, allowing precise quantification of preselected metabolites. | The coverage is limited, as it can only detect preselected metabolites, potentially missing important metabolites or metabolic pathways. |
| Untargeted Metabolomics | It offers broad coverage and can detect unknown or unexpected metabolites, making it suitable for discovering new biomarkers. | It has lower sensitivity and specificity, complex data analysis, difficulties in metabolite identification, and poor reproducibility of results. |
| Functional metabolomics | It can integrate multi-omics data to deeply analyze metabolic pathways and biomarkers, revealing dynamic metabolic changes within biological systems. | The technology is complex and expensive, and data analysis and metabolite identification are challenging, with functional validation often requiring additional experiments. |
| Spatial metabolomics | It provides comprehensive and rapid analysis of the relative abundance and spatial distribution of samples, allowing in situ reflection of spatial distribution information of substances. | Improvements are needed in terms of quantification capability, reproducibility, and resolution. |
| Single-cell metabolomics | It has high specificity, reproducibility, and sensitivity. | The contents are prone to loss, identification of metabolic molecules is challenging, and metabolites change rapidly within living cells. |
| Metabolic flux analysis | It detects subtle changes in biochemical pathways and offers high sensitivity, high specificity, and excellent reproducibility, with broad coverage of metabolites. | The experimental instruments are relatively expensive, and the sample processing and data measurement analysis are quite complex. |

**Table S4**

Advantages and disadvantages of the most widely applied chemometric algorithms in metabolomics [15, 105, 107].

| **Learning typology** | **Algorithms** | **Examples** | **Advantages** | **Disadvantages** | **Learning typology** |
| --- | --- | --- | --- | --- | --- |
| Unsupervised | Clustering | HCA, k-means | 1. Pattern recognition. 2. Initial dataset exploration. 3. Outlier identification. | 1. Poor management of multifactorial designs. 2. Non-optimal outputs. | Clustering |
|  | Dimensionality reduction | PCA, tSNE, NMF | 1. Definition of linear functions. 2. Proximity-based distribution. 3. Dataset complexity reduction. | 1. Require dimensionality optimization. 2. Loss of original overall variability. 3. Poor management of multifactorial designs | Dimensionality reduction |
| Supervised | Machine learning | PLS, OPLS, AMOPLS, SVM, KNN, RF | 1. Predicted discriminating modeling. 2. Statistical variation. 3. Discriminant markers identification (VIP). | 1. Random dataset splitting between training and test subsets. 2. VIP valorization in pairwise comparisons. 3. Require balanced designs. 4. Bad performance on high noise. | Machine learning |
|  | Deep learning | ANN, DNN | 1. Robust predicting modeling. 2. Simplified model interpretation. 3. Response optimization. | 1. Specific training for algorithm development. 2. Require ad hoc experimental designs. 3. Long modeling times and demanding informatic tools. | Deep learning |
